# Applying a deep learning-based sequence labeling approach to detect attributes of medical concepts in clinical text

**DOI:** 10.1186/s12911-019-0937-2

**Published:** 2019-12-05

**Authors:** Jun Xu, Zhiheng Li, Qiang Wei, Yonghui Wu, Yang Xiang, Hee-Jin Lee, Yaoyun Zhang, Stephen Wu, Hua Xu

**Affiliations:** 1 The University of Texas School of Biomedical Informatics, 7000 Fannin St Suite, Houston, TX 600 USA; 2 College of Computer Science and Technology, Dalian University of Technology, Dalian, China; 3 Departments of Health Outcomes and Policy, College of Medicine, University of Florida, Gainesville, Florida USA

**Keywords:** Information extraction, Natural language processing, Clinical notes

## Abstract

**Background:**

To detect attributes of medical concepts in clinical text, a traditional method often consists of two steps: named entity recognition of attributes and then relation classification between medical concepts and attributes. Here we present a novel solution, in which attribute detection of given concepts is converted into a sequence labeling problem, thus attribute entity recognition and relation classification are done simultaneously within one step.

**Methods:**

A neural architecture combining bidirectional Long Short-Term Memory networks and Conditional Random fields (Bi-LSTMs-CRF) was adopted to detect various medical concept-attribute pairs in an efficient way. We then compared our deep learning-based sequence labeling approach with traditional two-step systems for three different attribute detection tasks: disease-modifier, medication-signature, and lab test-value.

**Results:**

Our results show that the proposed method achieved higher accuracy than the traditional methods for all three medical concept-attribute detection tasks.

**Conclusions:**

This study demonstrates the efficacy of our sequence labeling approach using Bi-LSTM-CRFs on the attribute detection task, indicating its potential to speed up practical clinical NLP applications.

## Background

Clinical narratives are rich with patients’ clinical information such as disorders, medications, procedures and lab tests, which are critical for clinical and translational research using Electronic Health Records (EHRs). Clinical Natural Language Processing (NLP) has been a feasible way to extract and encode clinical information in notes. Various clinical NLP approaches and systems [1–4] have been developed to extract important medical entities from text and encode them into standard concepts in ontologies such as the UMLS (Unified Medical Language System). However, downstream clinical applications, such as clinical decision support systems, often require additional attribute information of medical concepts. For example, to provide accurate information about what drugs a patient has been on, a clinical NLP system needs to further extract the attribute information such as dosages, modes of administration, frequency of administration etc. in addition to the drug names. Many current clinical NLP systems/applications extract individual medical concepts without modeling their attributes or with limited types of attributes, partially due to the lack of general approaches to extract diverse types of attributes for different medical concepts.

A medical concept can be defined more precisely as an object and its allowable attributes. The object may be a disorder, drug, or lab test entity and attributes can be any of the sub-expressions describing the target concept. Attributes are prominent in clinical procedures and found in clinical notes frequently, and have surface forms that can be textual or numerical. Table 1 shows some important attributes of different medical concepts in clinical text. Disorder concepts always have attributes that indicate whether a disorder is absent, hypothetical, associated with someone else, conditional etc. Detailed medication data are often expressed with medication names and signature information about drug administration, such as dose, route, frequency, and duration. Laboratory analysis always originates numerical values for different lab tests.

**Table 1:** Medical concepts and their attributes

Concept	Attributes	Examples	Comments
Disorder	Negation, Severity, Body location, etc.	Denied any [chest pain]_Disorder_.	The disorder ‘chest pain’ has associated negation attribute “Denied” and body location attribute ‘chest’.
Medication	Dosage, Frequency, Mode, etc.	[insulin Lente]_Medication_ 12 units subcu q p.m.	The dosage attribute is ‘12 units’, the mode attribute is ‘subcu’ and the frequency attribute is ‘q p.m.’.
Lab Test	Lab value	[blood pressure]_LabTest_ 134/75 [URINE BLOOD]_LabTest_ - NEG	The ‘blood pressure’ has a numerical value ‘134/75’ and the ‘URINE BLOOD’ has a textual value ‘NEG’.

Recently, the Clinical NLP research community has increased its focus on the task of identifying attributes for medical concepts. For the past few years, a series of open challenges have been organized, which focused on not only identifying medical concepts but also their associated attributes from clinical narratives. The Third i2b2 Workshop focused on medication information extraction, which extracts the text corresponding to a medication along with other attributes that were experienced by the patients [5]. Attribute information to be targeted included dosages, modes of administration, frequency of administration, and the reason for administration. The ShARe/CLEF 2014 [6] and SemEval 2015 [7] organized open challenges on detecting disorder mentions (subtask 1) and identifying various attributes (subtask 2) for a given disorder, including negation, severity, body location etc. These challenges have greatly promoted clinical NLP research on attribute detection by building benchmark datasets and innovative methods.

The detection of medical concept attributes is typically mapped to the NLP tasks of named entity recognition (NER) and relation extraction. Many rule-based approaches have been proposed to extract the medical concept-associated attributes, relying on existing domain dictionaries and hand curated rules. MedLEE, perhaps the oldest and most well-known system, encodes contextual attributes such as negation, uncertainty and severity for indexed clinical conditions from clinical reports [8]. NegEx [9] and ConText [10] are other two widely used algorithms for determining contextual attributes for clinical concepts. ConText is an extension of the NegEx negation algorithm, which relies on trigger terms, pseudo-trigger terms, and termination terms to recognize negation, temporality, and experiencer attributes for clinical conditions. For medication information extraction, the earliest NLP system CLAPIT [11] extracted drug and its dosage information using rules. The system achieved an 86.7% exact match F-score. In the work of Gold et al. [12], a rule-based approach was proposed to extract drug attributes: dose, route, frequency and necessity. Another system, MedEx [13], is a rule-based sequence tagger that combined dictionary lookup, regular expression, and rule-based disambiguation components to label drug names and signatures in clinical text.

In addition, many high-performing systems in the above challenges used machine learning methods. The USyd system [14] achieved the best performances in the i2b2 2009 medication challenge, which incorporated both machine learning algorithms and rules engines. The system used a conditional random field (CRF) to identify medication and attribute entities, and a Support Vector Machine (SVM) determined whether a medication and an attribute were related or not. In the ShARe/CLEF 2014 and SemEval 2015 challenges, most participating systems also used machine learning-based approaches, coupled with related dictionaries, to extract disorder assertion attributes. For example, Team ezDI [15] detected disorder attributes in two steps: 1) used CRF to recognize attribute mentions 2) trained SVMs classifiers to relate the detected mentions with disorders.

These previous machine learning systems performed well on different attribute detection tasks, but this success was undercut by an important disadvantage. Most of them used a traditional two-step cascade approach: 1) Named Entity Recognition (NER), to recognize attribute entities from text; and 2) Relation extraction, to classify the relations between any pair of attribute and target concept entities. The two-step approach is built on different machine learning algorithms with massive human curated features, which is complicated. Moreover, to get better performance, in some systems, different models need to be built for each attribute separately. For example, Apache cTAKES treats the task of locating body sites and severity modifier as two different extraction problems and builds two different extraction modules [16]. In addition, the cascade approach may suffer from error propagation, so that any errors generated in the NER step may propagate to the step of relation classification.

In a previous shared task of “Adverse Drug Reaction (ADR) Extraction from Drug Labels” (2017 TAC-ADR), we proposed a sequence-labeling based approach to ADR attribute detection of drug mentions and it achieved superior performance (ranked No. 1 in the challenge) [17]. The proposed approach recognizes attribute ADRs and classifies their relations with the target drug in one step, after we transform the ADR attribute detection into a sequence-labeling problem. In this study, we extend this approach by modeling target concepts in a neural architecture that combines bidirectional LSTMs and conditional random fields (Bi-LSTM-CRF) [18] and apply it to clinical text to assess its generalizability to attribute extraction across different clinical entities including disorders, drugs, and lab tests. We conducted several experiments to compare our sequence labeling-based approach with traditional two-step extraction methods using three different corpora for disorders, medications and lab tests and our results show that the sequence labeling-based method achieved much better performance than traditional methods in all three tasks, indicating its utility to concept-attribute detection from clinical text.

## Materials and methods

### Tasks and datasets

In this study, we developed and evaluated our methods using three different attribute detection tasks:

#### ShARe-disorder

This task is to detect attributes of disorders in clinical documents. We used the ShARe corpus developed for the SemEval 2015 challenge task 14 [7], which is to recognize disorders and a set of attributes including: Negation indicator (NEG), Subject Class (SUB), Uncertainty indicator (UNC), Course class (COU), Severity class (SEV), Conditional indicator (CON), Generic indicator (GEN), and Body location (BDL). For simplicity, we removed all dis-joint disorder and attributes mentions and ignored the GEN detection task since more than 99% of disorders have no GEN attribute [7]. As the test dataset from this challenge was not released to public, we merged the training and development datasets (resulting in 431 de-identified clinical notes in total) and used them for this study.

#### i2b2-medication

This task is to detect signature attributes of drugs in clinical documents. We followed the 2009 i2b2 medication extraction challenge [19], which is to extract medications and their dosages (DOS), modes (MOD), frequencies (FRE), durations (DUR) and reasons (REA). We used the test corpus in the challenge, which consists of 251 discharge summaries with “silver” standard annotations collectively annotated by the challenge participants.

#### i2b2-LabTest

This task is to detect values (VAL) associated with lab tests mentioned in clinical documents. We leveraged the corpus used in the 2010 i2b2/VA shared task [20] to develop a newly annotated dataset for this task: we first extracted sentences containing lab test entities according to the original annotations in the challenge (2291 sentences in total) and then manually annotated values associated with each lab test mention (if any).

Table 2 shows the types of attributes for each of the three tasks, as well as statistics of the corpora used in this study.

**Table 2:** Concepts and attributes types included in this study, as well as their distribution in the corpora

Dataset	# Target Concepts	#Attribute Mentions	
ShARe-Disorder	17,368	NEG	3599
		SUB	191
		CON	927
		SEV	1286
		COU	901
		UNC	1348
		BDL	8053
i2b2-Medication	8251	DOS	3673
		MOD	2752
		FRE	3014
		DUR	259
		REA	537
i2b2-LabTest	7937	VAL	6644

### Traditional two-step approach (baseline system)

We developed a baseline system that uses the traditional two-step approach. It consists of two steps to identify attributes for a given medical concept. 1) Attribute entity recognition: NER task where named entities are attributes; we used a Bi-LSTM-CRF [18] as our sequence labeling algorithm, which has obtained state-of-the-art performance in different NER Tasks [3, 18]. 2) Attribute-concept relation extraction: We treated this task as that of relation classification between two entities. It was further divided into two tasks: candidate attribute-concept pair generation and classification. We generated all attribute-concept pairs within one sentence as candidates and then labeled them as positive or negative, based on the gold standard. We trained a binary classifier for each attribute to check if any relationship existed between an attribute mention and a concept. The first baseline system use the SVMs algorithm to classify candidate attribute-concept pairs, trained on both contextual and semantic features such as: words before, between, and after the attribute-concept pair; words inside attributes and concepts, and the relative position of attributes. The second baseline system combine a Bi-LSTM layer and a Softmax layer to classify candidate pairs [21]. To train this classifier, we use word embedding and position embedding as input features. Both of the embeddings are randomly initialized.

### Attribute detection by sequence labeling

**Fig. 1:**
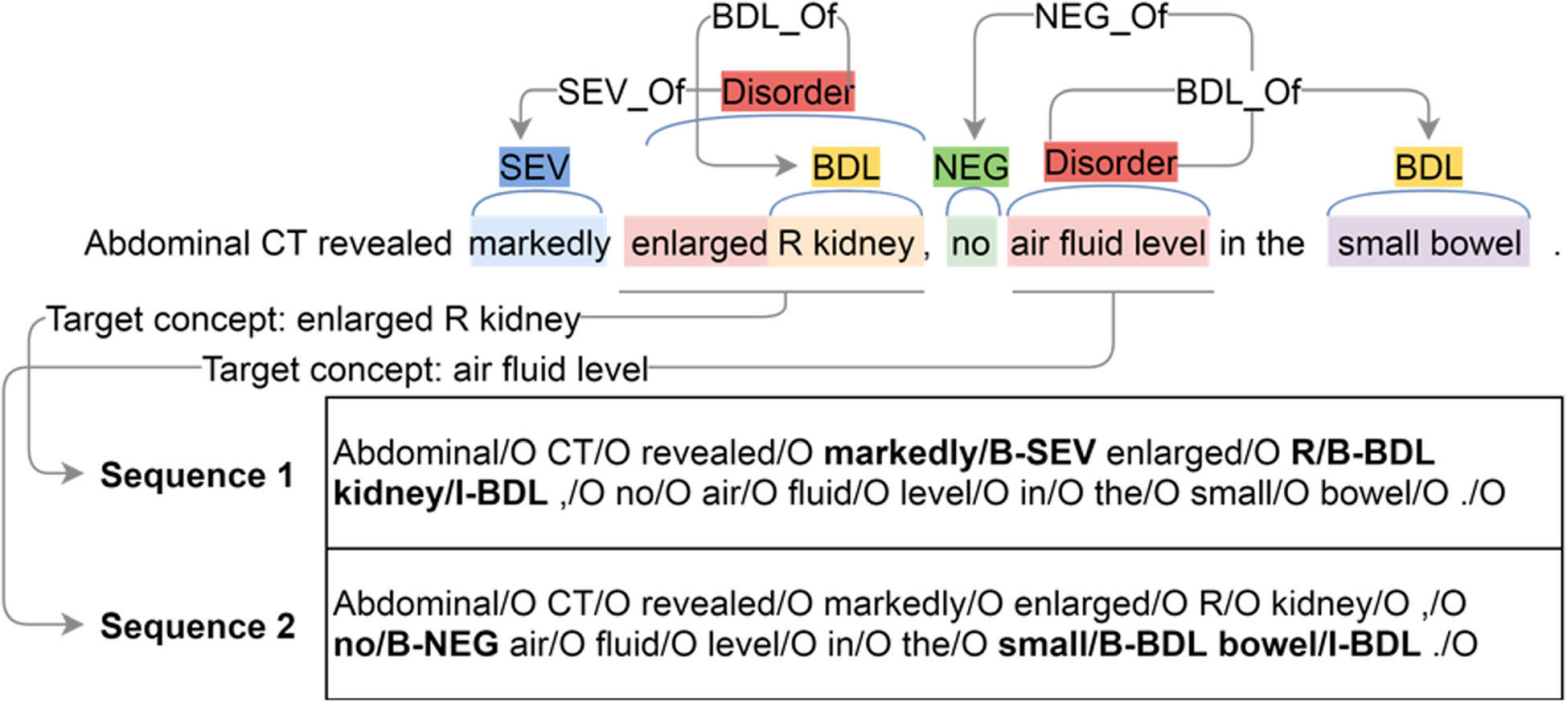
An illustration of the concept-focused sequence (CFS) transformation, where each separate sequence encodes all attributes for each target concept (Disorder)

Besides the issues of complexity and error propagation, the traditional two-step approach also faces a major problem, namely, omitted annotations of attribute entities. Attributes such as NEG and BDL may not be annotated in a gold standard corpus if they are not associated with a medical concept. For example, in the Fig. 1, ‘Abdominal’ is not annotated as a BDL entity in the ShARe-Disorder corpus. This makes it challenging to train an effective NER model for those attributes, and misses negative attribute-concept candidate pairs that are required to train an effective relation classifier. To address the above issues, we propose a novel sequence labeling approach for attribute detection, which identifies attribute entities and classifies relations in one-step. To address this issue, we proposed a new transformation method in the TAC ADR detection challenge and converted it into a sequence labeling problem [17]. Here we extend this approach to make it generalizable for any types of clinical concepts of interests.

Taking an example of disorder-modifier extraction task (as shown in Fig. 1), one sentence may have multiple target concepts (i.e., disorders) mentioned. In this case, we will produce multiple training samples (named “concept-focused sequences” - CFS) from the same sentence - one for each target concept. For each CFS, attributes that are associated with the target concept are labeled using the BIO scheme (the Beginning, Inside, or Outside of a named entity). For the example in Fig. 1, there are two disorder concepts: “enlarged R kidney” and “air fluid level”, each of which will generate a CFS for training. In the CFS for “enlarged R kidney”, only attributes that are associated with it (i.e., “markedly” and “R kidney”) are labeled with B or I tags. Attributes associated with “air fluid level” (i.e., “no” and “small bowel”) are labeled with the O tag in the CFS of “enlarged R kidney”. With such a transformation, the task is to label a CFS to identify attributes associated with a known target concept.

To model the target concept information alongside a CFS, we slightly modified the Bi-LSTM-CRF architecture, by concatenating the vector representations of the target concept with the vector representations of individual words. We used “Target” and “NotTarget” tags to distinguish the target concept from other non-target concepts and embeddings of each tag was randomly initialized and learned directly from the data during the training of the model.

### Experiments and evaluation

Initial experiments showed that pre-trained word embeddings did not improve overall performance much. Therefore, we initialized our word embeddings lookup table randomly in all our experiments. In the sequence labeling approach, the dimension of the semantic tag embeddings for target concept was set to 10. Tuning this dimension did not significantly affect model performance. For both methods, their Bi-LSTM-CRF models used the same parameters: a word embedding size of 50; a character embedding size of 25; a word-level hidden LSTM layer size of 100 and a character-level hidden LSTM layer size of 25; stochastic gradient descent with a learning rate of 0.005; dropout with a probability of 0.5.

Our evaluation is based on correctness in assigning attribute mentions to the given medical concepts. Here, we use the standard precision (P), recall (R) and F-measure under strict criteria as our evaluation metrics. We align the gold standard and the system output using the given concepts (name and offset). Note that in these results, an attribute mention associated with multiple concepts will be calculated multiple times - this differs slightly from traditional NER tasks, in which entities can only be calculated once. We also adopt accuracy (Acc) to evaluate the ability of detecting specific attribute (including null) on concept level, defined as:

$$ Acc=\frac{N_{correct\_ predict}}{N} $$

Where, N is the total number of gold standard concepts, N_correct_predict_ is the number of concepts, and attributes are strictly matched. For each task, we conducted 10-fold cross validation and reported micro-averages for each attribute type.

We evaluated our system without the use of external data or knowledge bases. The attributes we have explored are not interchangeable in their meanings or linguistic patterns (e.g., compare concept negation to medication reason). So external data sources would have inconsistent effects on the task, and the generalizability of our methods would be less clear. Thus, we use only features that are learned directly from the data in our experiments.

## Results

Tables 3, 4 and 5 show our results on attribute detection for disorders, medications, and lab tests, respectively. On the three datasets, the proposed sequence labeling approach using Bi-LSTM-CRF model greatly outperformed the traditional two-step approaches. On the detection of disorders attributes, as shown in Table 3, the F1 scores for COU and UNC detection were much lower than other attributes. On medication attribute detection, compared to the baseline systems, the sequence labeling approach achieved lower F-scores but higher accuracy on FRE, DUR and REA detection. The VAL attribute detection for lab tests was the easiest task, and the sequence labeling approach achieved an F1 of 0.9554. We show the state-of-the-art Usyd system [14] for reference, though it is unfair to compare our system with USyd directly, since our system takes gold medications as inputs while USyd was an end-to-end system and trained with extra annotated corpora.

**Table 3:** The overall performance of different approaches on the share-disorder dataset in detecting 7 attributes of given disorders: negation (neg), subject (sub), conditional (con), severity (sev), course (cou), uncertainty (unc), body location (bdl). best results are shown in boldface

Attribute		NEG	SUB	CON	SEV	COU	UNC	BDL
1.1.1.Baseline (Bi-LSTM-CRF + SVM)	Acc.	0.9323	0.9929	0.9669	0.9655	0.9576	0.9445	0.7524
	P	0.7931	0.7374	0.6990	0.6421	0.5068	0.4091	0.5887
	R	0.7768	0.6348	0.5987	0.7568	0.6437	0.4172	0.7516
	F	0.7849	0.6822	0.6449	0.6948	0.5671	0.4131	0.6602
1.1.1.Baseline (Bi-LSTM-CRF + Bi-LSTM)	Acc.	0.9146	0.9900	0.9632	0.9707	0.9597	0.9308	0.7859
	P	0.8387	0.8158	0.7872	0.7609	0.6340	0.4380	0.7218
	R	0.7277	0.5391	0.6054	0.8213	0.6322	0.3819	0.784
	F	0.7793	0.6492	0.6844	0.7900	0.6331	0.4080	0.7516
1.1.1.Sequence Labeling	Acc.	**0.9542**	**0.9937**	**0.9718**	**0.9817**	**0.9697**	**0.955**	**0.8695**
	P	0.8142	0.8222	0.7583	0.7812	0.6150	0.4854	0.7887
	R	0.8310	0.6435	0.6682	0.8859	0.7529	0.4393	0.7991
	F	**0.8225**	**0.7220**	**0.7104**	**0.8302**	**0.6770**	**0.4612**	**0.7939**

**Table 4:** The overall performance of different approaches on the i2b2-medication dataset in detecting 5 attributes of given medications: dosage (dos), mode (mod), frequency (fre), duration (dur), reason (rea). best results are shown in boldface

Attribute		DOS	MOD	FRE	DUR	REA
Baseline (Bi-LSTM-CRF + SVM)	Acc.	0.9201	0.9584	0.9353	0.9783	0.9473
	P	0.8794	0.9110	0.8762	0.5945	0.5373
	R	0.9292	0.9597	0.9390	0.6680	0.6704
	F	0.9036	0.9347	0.9065	0.6291	**0.5965**
Baseline (Bi-LSTM-CRF + Bi-LSTM)	Acc.	0.9250	0.9559	0.9302	0.9680	0.9269
	P	0.9305	0.9372	0.9198	0.6168	0.5984
	R	0.9434	0.9658	0.9399	0.6525	0.5717
	F	0.9369	0.9513	**0.9298**	**0.6341**	0.5848
Sequence Labeling	Acc.	**0.9573**	**0.9807**	**0.9556**	**0.9802**	**0.9589**
	P	0.9728	0.9773	0.9503	0.7785	0.7409
	R	0.9159	0.9528	0.9078	0.4479	0.4953
	F	**0.9435**	**0.9649**	0.9286	0.5686	0.5938
Usyd [14]	P	0.9189	0.9073	0.9142	0.5604	0.6687
	R	0.8678	0.8915	0.8795	0.3709	0.3319
	F	0.8926	0.8994	0.8965	0.4464	0.4436

**Table 5:** The overall performance of different approaches on the i2b2-labtest dataset in detecting values (val) of given lab tests. Best results are shown in boldface

Attribute		VAL
Baseline (Bi-LSTM+SVM)	Acc.	0.4415
	P	0.7160
	R	0.4193
	F	0.5289
Baseline (Bi-LSTM+Bi-LSTM)	Acc.	0.8993
	P	0.9248
	R	0.9288
	F	0.9268
Sequence Labeling	Acc.	**0.9456**
	P	0.9526
	R	0.9582
	F	**0.9554**

## Discussion

In this paper, we investigated a sequence-labeling based approach for detecting various attributes of different medical concepts. The proposed approach transforms the attribute detection of given concepts into a sequence-labeling problem and adopts a neural architecture that combined bidirectional LSTMs and CRF as sequence labeling algorithm. It recognizes attribute entities and classifies their relations with the target concept in one-step. The experiments on three attribute detection tasks show good performance of our proposed method.

A few specific types of attributes appear to be particularly difficult to detect; for example, the F1 of disorder uncertainties (UNC), medication durations (DUR), and medication reasons (REA) were all lower than 0.6. This could be due to diversity of the surface forms and low frequency of these attributes in our datasets. For example, in the i2b2-Medication dataset, there are 259 DUR entities in total, which is relatively small for training a machine learning model to recognize named entities without extra knowledge. In addition, we found that the data for the REA and DUR attribute relation classifiers were heavily biased towards positive samples. This bias may make the binary classifiers tend to relate the given medication with the detected DUR or REA attribute entities.

For each of the 13 attributes in Tables 3, 4 and 5, we randomly selected ten errors by our system for analysis. After manually checking these 130 errors, we classified the errors into the following five types: 1) Matching partially (26/130): the boundaries of the attribute entity do not perfectly match. 2) Relating with wrong target concept (21/130): the error where the system recognized an attribute entity and related it with wrong target concept. 3) Missing one of attribute cues (5/130): the attribute of the target concept has more than one cue. However, the system only finds one of them. 4) Annotation errors (13/130). 5) Other diverse, but unclear reasons, including unseen samples (65/130). For example, “precath” is not extracted as a MOD from the sentence “[Mucomyst] medication precath with good effect”. A potential reason may be that the use of “precath” is unusual. Table 6 lists examples for each type of errors.

**Table 6:** Examples of attribute detection errors

Error Type	Frequency	Example			
		*Sentence & Target Concept*	*Attribute*	*Gold Standard*	*System Prediction*
Matching partially	26/130	… were negative for [infection]_disorder_	NEG	negative	negative for
Relating with wrong target concept	21/130	… multiple small [collections of blood] in your head.	BDL	head	blood, head
Missing one of attribute cues	5/130	[Ultralente]_medication_ 14 mg q.a.m., 4 mg	DOS	14 mg; 4 mg	14 mg
Annotation errors	13/130	Father died from [CHF]_disorder_ at 54	SUB		Father
Others	65/130	[Mucomyst]_medication_ precath with good effect	MOD	precath	

This study has several limitations. First, our Bi-LSTM-CRF system was not fully optimized for the problem setting. For example, we did not use pretrained embeddings or external knowledge bases and we did not consider alternative deep learning architectures. In the future we will investigate existing domain knowledges and integrate them as features into our models to further reduce recognition errors discussed in the error analysis. Moreover, as contextual language representation has achieved many successes in NLP tasks [22, 23], we will explore the usage of novel contextual word embeddings to replace randomly initialized word embeddings and pre-train them with external clinical corpora. Second, while we did achieve state-of-the-art performance on all three tasks, the generalizability of our approaches need further validation, as data sources used here were limited to a single corpus for each type of concept-attribute. Furthermore, we also suffered from the lack of sufficient annotated data for specific types of attributes, thus optimal performance was not achieved.

## Conclusions

In this study, we proposed a sequence-labeling based approach for detecting attributes of different medical concepts, which recognizes attribute entities and classifies their relations with the target concept in one step. Our experimental results show that the proposed technique is highly effective. This study demonstrates the efficacy of our sequence labeling approach using Bi-LSTM-CRFs on the attribute detection task. The proposed deep learning-based architecture provides a simple unified solution for detecting attributes for given concepts without using any external data or knowledge bases, thus streamlining applications in practical clinical NLP systems.

## Data Availability

Not applicable.

## Abbreviations

ADR: Adverse drug reaction
BDL: Body location
Bi-LSTM-CRF: Bidirectional long short-term memory and conditional random field
CFS: Concept-focused sequence
CON: Conditional indicator
COU: Course class
CRF: Conditional random field
DOS: Dosage
DUR: Duration
EHRs: Electronic health records
FRE: Frequency
GEN: Generic indicator
MOD: Mode
NEG: Negation indicator
NER: Named entity recognition
NLP: Natural language processing
REA: Reasons
SEV: Severity class
SUB: Subject class
SVM: Support vector machine
UMLS: Unified medical language system
UNC: Uncertainty indicator
VAL: Value
